# Diffuse Hepatic Calcifications in a Transfusion-Dependent Patient with Beta-Thalassemia: A Case Report

**Published:** 2013-09

**Authors:** Forough Saki, Mohammad Reza Bordbar, Mohammad Hadi Imanieh, Mehran Karimi

**Affiliations:** 1 Department of Pediatric Endocrinology, Student Research Center, Shiraz University of Medical Sciences, Shiraz, Iran;; 2 Hematology Research Center, Nemazee Hospital, Shiraz University of Medical Sciences, Shiraz, Iran;; 3 Gastroenterohepatology Research Center, Department of Pediatric Gastroenterology, Nemazee Hospital, Shiraz University of Medical Sciences, Shiraz, Iran

**Keywords:** Beta-Thalassemia major, Hepatic, Calcification, Hypoparathyroidism

## Abstract

Hepatic calcification is usually associated with infectious, vascular, or neoplastic processes in the liver. We report the first case of beta-thalassemia major with isolated diffuse hepatic calcification in a 23 year old woman, who had been transfusion-dependent since the age of 6 months. She was referred to our center with a chief complaint of abdominal pain. Computed tomography scan of the abdomen revealed diffuse hepatic calcification in the right, left, and caudate lobes of the liver. Her medical history disclosed hypoparathyroidism as well as chronic hepatitis C virus infection, which was successfully treated but led to early micronodular cirrhosis on liver biopsy. Other studies done to search for the cause of hepatic calcification failed to reveal any abnormalities. We suspect that hypoparathyroidism caused liver calcification, and should be, therefore, considered in the differential diagnosis of hepatic calcification if other causative factors have been ruled out.

## Introduction

Patients with beta-thalassemia are confronted with various complications mainly due to iron overload. Hypoparathyroidism is one of the most prevalent endocrine complications of thalassemia major, leading to hypocalcemia, tetanus, and seizure.^1^ Isolated intracerebral calcification due to hypoparathyroidism has been reported previously.^2^^-^^4^ Isolated hepatic calcification, however, is an infrequent finding that has not been reported in patients with thalassemia. Here we report the first case of hypoparathyroidism in a beta-thalassemia major patient, who presented with isolated diffuse hepatic calcification.

## Case Report

**Figure 1:**
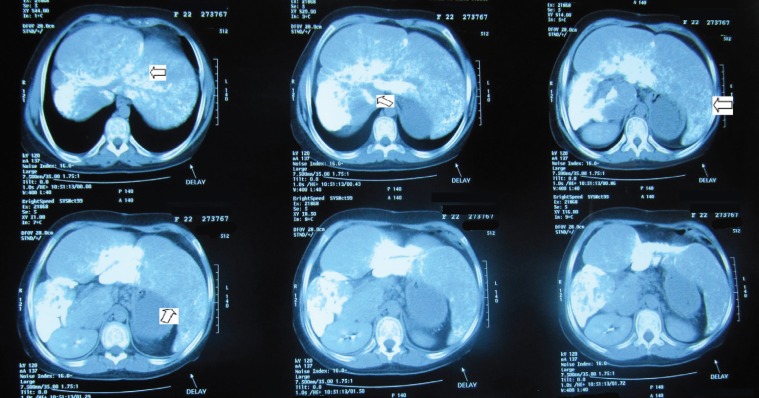
This is abdominal spiral CT-scan with intravenous and oral contrast of the patient, who had beta-thalassemia major and hypoparathyroidism. The arrows point at the hypertrophy of the left and caudate lobes of the liver in addition to severe calcification in the right, left, and caudate lobes

**Figure 2:**
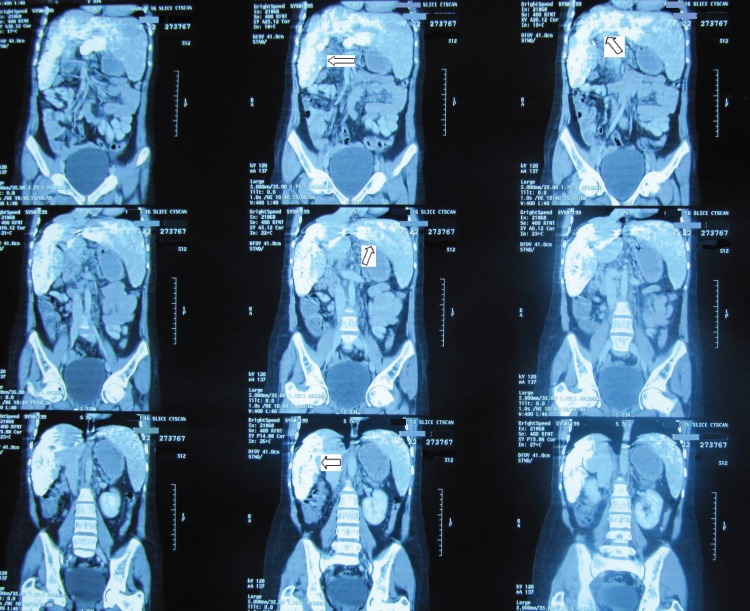
The arrows show that the densest parts are the posterior aspect of the segments 4, 2, and 3 as well as the right lobe. Inhomogeneous parenchymal density, representative of liver parenchymal damage, and dilated portal vein with multiple collateral veins in the epigastrium, due to portal hypertension, are also evident

A 23-year-old woman with beta-thalassemia major and transfusion dependence presented with abdominal pain of 3 weeks’ duration. Some workup was done to find out the cause of the abdominal pain. Incidentally, diffuse calcification of the liver was found in abdominopelvic sonography. This finding was confirmed with abdominopelvic CT, which also revealed that the calcification was confined to the liver and that the kidneys or other internal organs were not involved (figures 1 and 2). After 2 weeks, the abdominal pain was subsided with Omeprazol (20 mg/day) and conservative therapy. It seems that the abdominal pain was not related to the liver calcification and was an incidental finding.

The diagnosis of beta-thalassemia major had been confirmed when the patient was 6 months old based on complete blood count and hemoglobin electrophoresis. Since then, she has been on regular transfusion every 2-3 weeks. She underwent splenectomy at the age of 6 years and has taken penicillin V (250 mg orally twice per day) as prophylaxis ever since. The diagnosis of hypoparathyroidism was made 8 years prior to her referral to us on the basis of low serum calcium (Ca=5.6 mg/dl), high serum phosphorus (Ph=9.6 mg/dl), and low intact parathyroid hormone levels (PTH=5 pg/ml), for which she has taken Calcitriol and calcium carbonate to maintain calcium and phosphorus hemostasis. On follow-up, serum calcium was in the range of 8-10 mg/dl and serum phosphorous was in the range of 4-6 mg/dl. Bone mineral densitometry revealed severe osteoporotic changes in the lumbar vertebrae (Z-score -2.8) and femoral neck (Z-score -0.8), for which she has taken alendronate (70 mg orally) weekly. Because she had moderate left ventricular dysfunction on echocardiography, a cardiologist prescribed captopril (25 mg orally) and furosemide (20 mg orally per day). She had a positive serological finding for hepatitis-C virus (HCV), which was confirmed by polymerase chain reaction (PCR) when she was 13 years old. She was successfully treated with pegylated interferon and Ribavirin for 2 consecutive years due to persistent HCV infection, and her PCR results for HCV were negative at that time she was referred to us. Liver biopsy at that time revealed early micronodular cirrhosis, but no calcification was found. She received iron chelation treatment with subcutaneous injections of Deferoxamine (50 mg/kg) every other night and daily Deferiprone (75 mg/kg orally 3 times per day), and her most recent serum ferritin concentration was 495 ng/mL.

Because of the patient’s abdominal pain, extensive workup-including abdominal sonography and abdominal spiral computed tomography scan (CT scan) with intravenous and oral contrast were performed. This revealed hypertrophy of the left and caudate lobes of the liver in addition to severe calcification in the right, left, and caudate lobes. The greatest densities were located in the posterior aspect of segments 4, 2, and 3 as well as the right lobe. Non-homogenous parenchymal density, representative of liver parenchymal damage, and portal vein dilation with multiple collateral veins in the epigastrium, due to portal hypertension, were also evident (figure 1). Other studies, including brain CT scan and ophthalmologic examination for cataracts, revealed no abnormal metastatic calcifications. The results of other paraclinical tests-including liver and renal function tests, fasting blood glucose, calcium, phosphorus, prothrombin and partial thromboplastin times, thyroid function, alfa feto protein, beta-HCG level, tuberculin skin test, serology for brucella infection, hepatitis B and C, anti-cytomegalovirus IgM and IgG, human immunodeficiency virus, serology for toxoplasma infection, and workup for hydatid cyst and amebic and fungal infections, revealed no abnormalities. 

The patient was followed up closely, and her abdominal pain subsided spontaneously. However, she was incidentally found to have isolated hepatic calcification, which may have been due to hypoparathyroidism.

## Discussion

Hepatic calcification is a rare event which usually occurs as a result of inflammatory conditions. The main causes of hepatic calcification are infections-e.g. tuberculosis, histoplasmosis, brucellosis, schistosomiasis, hydatid cyst, cytomegalovirus, toxoplasmosis, *Pneumocystis carinii* infection, chronic amebic or pyogenic abscess, and chronic granulomatous disease of childhood. Vascular problems-including hepatic artery aneurysm, portal vein thrombosis, and hematoma as well as neoplastic processes such as hemangioma, hepatocellular adenoma and carcinoma, infantile hemangioendothelioma, cholangiocarcinoma, hepatoblastoma, and metastatic tumors of the liver represent the remaining etiologies.^5^ Diffuse hepatic calcification is seen even more rarely and the differential diagnosis is narrower. It usually occurs after ischemic insult in patients with end-stage renal disease on hemodialysis and as a sequella of shock liver.^6^^,^^7^

To find out the cause of diffuse hepatic calcification, we should rule out other differential diagnosis in each case. We evaluated liver and renal function tests, fasting blood glucose, calcium, phosphorus, prothrombin and partial thromboplastin times, thyroid function, tuberculin skin test, serology for brucella infection, hepatitis B and C, anti-cytomegalovirus IgM and IgG, human immunodeficiency virus, serology for toxoplasma infection, and workup for hydatid cyst and amebic and fungal infections to rule out renal failure. Additionally, we evaluated infections such as tuberculosis, histoplasmosis, brucellosis, schistosomiasis, hydatid cyst, cytomegalovirus, toxoplasmosis, *Pneumocystis carinii* infection, chronic amebic or pyogenic abscess, and chronic granulomatous disease of childhood. All the tests were normal and showed no hint of infection. Also, level of alfa feto protein, serum BHCG, abdominal sonography, portal and hepatic vein Doppler sonography, and abdominal spiral CT scan with intravenous and oral contrast were conducted and revealed no clue for vascular problems-including portal vein thrombosis and hematoma-as well as neoplastic processes such as hemangioma, hepatocellular adenoma and carcinoma, infantile hemangioendothelioma, cholangiocarcinoma, hepatoblastoma, and metastatic tumors of the liver. Paraclinical tests demonstrated that the patients had low calcium, high phosphate, and low PTH levels; a diagnosis of hypoparathyroidism was, therefore, established for the patient.

In thalassemia major patients, iron deposition (secondary to chronic anemia) in the parathyroid gland causes hypoparathyroidism and it suppresses the parathyroid hormone secretion. The lab findings in hypoparathyroidism are hypocalcemia, hyperphosphatemia, normal or low serum level of alkaline phosphatase, and normal or low serum level of parathyroid hormone.^1^ Hypoparathyroidism is associated with metastatic calcification in the central nervous system, mainly in the basal ganglia and rarely outside the extrapyramidal system;^2^^-^^4^ be that as it may, in all previous studies - hepatic calcification has never been reported in hypoparathyroidism. The pathogenesis of metastatic calcification in hypoparathyroidism might be due to decreased bone reservoir for the absorption of calcium and phosphate from the intestine, which causes extra osseous calcification.^8^

Although our patient had a history of hepatitis C infection 10 years earlier, she had never experienced fulminant hepatitis or hepatic failure in the course of the infection. She was successfully treated with pegylated interferon and Ribavirin for 2 consecutive years due to persistent HCV infection, and her PCR results for HCV were negative at that time of her referral to us. Because there was no infectious, vascular, or neoplastic process that could explain the liver calcification in the patient, we concluded that hypoparathyroidism remained the only likely explanation for this phenomenon. The mechanism for hepatic calcification in a patient with thalassemia major and hypoparathyroidism may be altered calcium and phosphorus hemostasis due to increased intestinal absorption and decreased bone deposition, leading to metastatic calcification, maybe superimposed on a damaged liver parenchyma affected by hemochromatosis and post-HCV hepatic cirrhosis.^8^

To the best of our knowledge, this is the first case report of beta-thalassemia major with diffuse hepatic calcification. It seems reasonable to consider hypoparathyroidism as a causal factor if other studies fail to reveal any abnormalities. Consequently, hypoparathyroidism is one of the possible, and not the sole, explanations of the problem.

## Conclusion

Diffuse hepatic calcification is a rare occurrence in patients with thalassemia. Although inflammatory conditions are the most common cause of hepatic calcification, hypoparathyroidism should also be considered in the differential diagnosis. 
